# Goldene: An Anisotropic Metallic Monolayer with Remarkable Stability and Rigidity and Low Lattice Thermal Conductivity

**DOI:** 10.3390/ma17112653

**Published:** 2024-05-31

**Authors:** Bohayra Mortazavi

**Affiliations:** 1 Institute of Photonics, Department of Mathematics and Physics, Leibniz Universität Hannover, Welfengarten 1A, 30167 Hannover, Germany; bohayra.mortazavi@gmail.com; 2 Cluster of Excellence PhoenixD, Leibniz Universität Hannover, Welfengarten 1A, 30167 Hannover, Germany

**Keywords:** goldene, machine learning, tensile strength, lattice thermal conductivity, metallic monolayer

## Abstract

In a recent breakthrough in the field of two-dimensional (2D) nanomaterials, the first synthesis of a single-atom-thick gold lattice of goldene has been reported through an innovative wet chemical removal of Ti_3_C_2_ from the layered Ti_3_AuC_2_. Inspired by this advancement, in this communication and for the first time, a comprehensive first-principles investigation using a combination of density functional theory (DFT) and machine learning interatomic potential (MLIP) calculations has been conducted to delve into the stability, electronic, mechanical and thermal properties of the single-layer and free-standing goldene. The presented results confirm thermal stability at 700 K as well as remarkable dynamical stability of the stress-free and strained goldene monolayer. At the ground state, the elastic modulus and tensile strength of the goldene monolayer are predicted to be over 226 and 12 GPa, respectively. Through validated MLIP-based molecular dynamics calculations, it is found that at room temperature, the goldene nanosheet can exhibit anisotropic tensile strength over 9 GPa and a low lattice thermal conductivity around 10 ± 2 W/(m.K), respectively. We finally show that the native metallic nature of the goldene monolayer stays intact under large tensile strains. The combined insights from DFT and MLIP-based results provide a comprehensive understanding of the stability, mechanical, thermal and electronic properties of goldene nanosheets.

## 1. Introduction

Monoelemental nanomembranes, such as graphene [1,2,3], borophene [4,5], phosphorene [6,7], silicene [8], germanene and stanene [9], have emerged as pivotal nanomaterials in the realm of nanotechnology [10], with highly promising properties for diverse groundbreaking applications across various domains. Graphene shows exceptional mechanical strength [11], ultrahigh thermal conductivity [12,13] and appealing electronic and optical properties [14,15,16,17]. While silicene, germanene and stanene monolayers also have hexagonal unit cells and exhibit a semimetallic electronic nature to graphene, because of their buckled structure and heavier atoms, they show considerably lower elastic modulus, tensile strength and lattice thermal conductivity [18] than graphene. Borophene monolayers can appear in diverse completely flat [5] or buckled [4] configurations, but they all demonstrate a metallic electronic nature and are accordingly decent electric conductors [19,20,21]. According to various theoretical studies [22,23], borophene nanosheets have been predicted to show ultrahigh energy storage capacities for applications in various rechargeable metal-ion batteries. Unlike the aforementioned monoelemental lattices, phosphorene [6,7], with a corrugated atomic configuration, exhibits a semiconducting electronic character with remarkably high carrier mobilities, boosting its application in nanoelectronics, optoelectronics and various energy storage and conversion systems [7,24,25]. In a recent breakthrough in the synthesis of monoelemental nanomembranes, goldene [26], the monolayer formed of gold atoms, has been exfoliated through an innovative wet chemical removal of Ti_3_C_2_ from the layered Ti_3_AuC_2_. From a practical standpoint, assessing the various physical properties of the goldene nanosheets is crucial to determine their suitability across diverse applications. To meet this need, for the first time, we employed a synergistic approach, combining first-principles density functional theory (DFT) calculations with classical modeling based on machine learning interatomic potentials (MLIPs). This comprehensive analysis allowed us to evaluate the stability, electronic, mechanical and phononic properties of the pristine and suspended goldene monolayer.

## 2. Computational Methods

In this communication, Vienna Ab initio Simulation Package (VASP) [27] was utilized, employing the generalized gradient approximation (GGA) and Perdew–Burke–Ernzerhof (PBE) exchange-correlation functional [28] and projector augmented-wave (PAW) [29] method for different computations, including structural optimizations, ab initio molecular dynamics (AIMD) simulations, analyses of mechanical properties and electronic structure calculations. The plane wave cutoff energy was set as 300 eV with self-consistency convergence criteria of 10^−6^ eV, adopting a 15 × 15 × 1 K-point Monkhorst Pack mesh [30]. For the analyses of electronic band structures, the plane wave cutoff energy was increased to 400 eV, adopting also a finer K-point grid (35 points in every path). In order to achieve geometry optimization and obtain stress-free structures, adjustments were made to atomic positions and lattice sizes by using the conjugate gradient algorithm until the Hellman–Feynman forces on each atom were reduced to below 0.001 eV/Å on each atom. The nanosheets were positioned in the XY plane, and in order to remove interactions between neighboring cells in the Z direction, around 14 Å of vacuum space was introduced. Density functional perturbation theory (DFPT) calculations were carried out with the VASP package to derive phonon dispersions employing the PHONOPY code [31], based on the 9 × 5 × 1 supercell (90 atoms). Moment tensor potential (MTP) [32] formalism was used to investigate the structural, dynamical, thermal and mechanical properties. The training dataset was prepared by the AIMD calculations over the stress-free and stretched supercells with 36 atoms under varying temperatures, using the same methodology as our recent works [33,34], adopting a time step of 1 fs, NVT ensemble and 2 × 2 × 1 Monkhorst Pack mesh. An MTP with a cutoff distance of 5.0 Å was trained using the two-step passive training approach [35]. The phonon dispersion relation was obtained by utilizing the trained MTP, employing 9 × 5 × 1 supercells and applying the small displacement technique from the PHONOPY package [31], as detailed in our previous study [36]. In the data availability section, the energy-minimized lattice, fitted MTP and corresponding training data are fully given. VESTA [37] and OVITO [38] free packages were employed to illustrate the atomic structures. We utilized the LAMMPS package [39] to examine thermal and mechanical properties based on the trained MTP, with a time step of 0.5 fs. We assumed a fixed thickness of 3.32 Å for the goldene monolayer, according to the van der Waals (vdW) diameter of the gold atoms. Non-equilibrium molecular dynamics (NEMD) simulations on the basis of the trained MTP were carried out to evaluate the length-dependent lattice thermal conductivity at 300 K, using the same approach as that detailed in our previous study [40].

## 3. Results and Discussions

**Figure 1:**
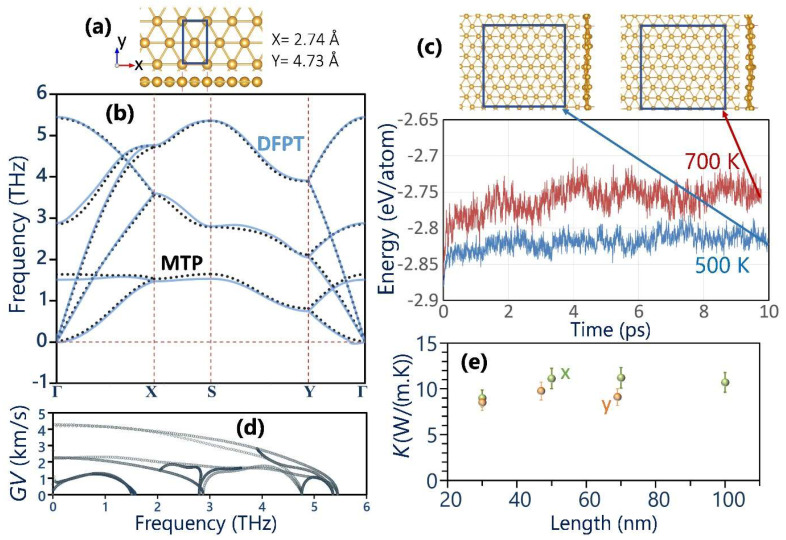
(**a**) Top and side views of crystal structures of the goldene monolayer. (**b**) Phonon dispersion relation predicted by the DFPT (continuous lines) and MTP (dotted line). (**c**) The AIMD analysis of the thermal stability at 500 and 700 K alongside atomic configurations after around 10 ps of calculations. (**d**) Phonon group velocity (GV) of the goldene monolayer. (**e**) Length-dependent lattice thermal conductivity of the single-layer goldene along x and y directions at room temperature.

Figure 1a illustrates the top and side views of the crystal structure of the goldene monolayer, which features a topologically flat arrangement and a triangular lattice structure, similar to α-beryllene [41]. In our work, the Au-Au bond length in the stress-free lattice was predicted to be 2.742 Å, which is close to the experimentally measured and theoretically calculated values of 2.62  and 2.735 Å, respectively, reported in the original experimental work [26]. Investigating the dynamical instability of the goldene monolayer based on phonon dispersion relation provides very insightful information. In this case, we compare the results obtained from the DFPT method with those from the MTP approach to validate the accuracy of the developed classical model. The phonon dispersion relations obtained from both methods are presented in Figure 1b, not only confirming the dynamical stability of the suspended goldene monolayer with the fully flat configuration but also showcasing the impressive precision of the MTP classical model in analyzing the phononic response of this novel nanosheet. Similar to other 2D materials and exactly like graphene, the suspended goldene exhibits three acoustic modes originating from the Γ point, and due to its primitive cell consisting of only two atoms, three optical modes also exist. However, in contrast to graphene, which features a frequency range extending to around 50 THz, the frequency range of the goldene monolayer is significantly narrower by nearly an order of magnitude. This observation underscores the suppressed group velocity for phonons in the goldene monolayer, attributable to the higher mass of gold atoms as compared to their carbon counterparts, potentially resulting in a much lower lattice thermal transport. Furthermore, assessing the thermal stability based on AIMD calculations reveals interesting insights. Figure 1c illustrates the evolution of the goldene monolayer potential energy per atom over AIMD simulation time. Remarkably, at both temperatures of 500 and 700 K, the goldene nanomembranes could exhibit robust integrity. The presented results highlight that goldene nanosheets can demonstrate decent flexibility and stability, indicating promising potential for practical applications. The predicted phonon group velocity of the goldene monolayer is shown in Figure 1d. The in-plane longitudinal acoustic phonon modes display the widest dispersions; as such, they present the highest velocities around 4.25 km/s, almost two-fold larger than that of their transverse acoustic counterparts. These values are around five- and two-fold lower than the corresponding values of graphene [36] and the quasi-hexagonal C_60_ monolayer [42], suggesting the low lattice thermal conductivity of the goldene system. To better evaluate this aspect, MLIPs can provide a highly accurate understanding [43,44,45,46,47,48]. In Figure 1f, the MTP-based NEMD predictions for the length effects on the room temperature phononic thermal conductivity of the goldene nanosheet along the x and y directions are plotted. For the samples along the x direction, the NEMD calculations were conducted for systems with up to 100 nm, and it appears that the lattice thermal conductivity is well converged for the system with a length around 50 nm, consistent with previously observed low phonon group velocities. For the heat transport along the y direction, we obtained close values for the lattice thermal conductivity of systems with lengths of 47 and 70 nm. Based on the presented results, the room temperature diffusive lattice thermal conductivity of the goldene monolayer is estimated to be 11.2 and 9.5 ± 1 W/(m.K) along the x and y directions, respectively, which, as discussed earlier, is rather low.

**Figure 2:**
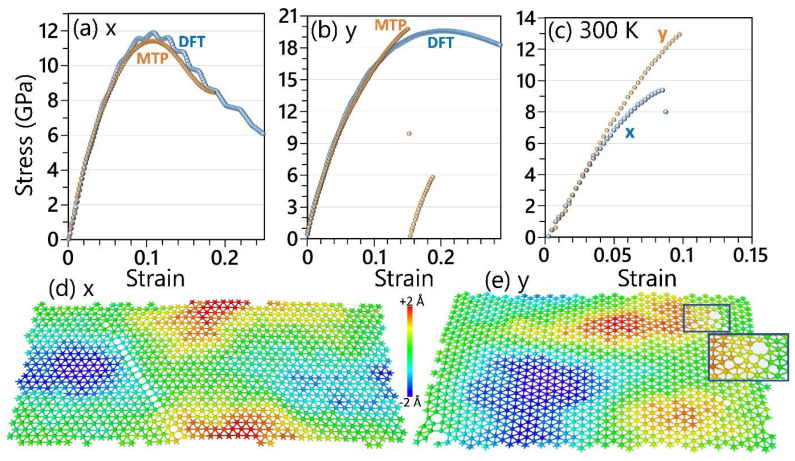
(**a**,**b**) Predicted uniaxial stress–strain responses of the pristine goldene by the MTP and DFT models loaded along x and y directions, respectively. (**c**) Uniaxial stress–strain response at 300 K by the MTP model. (**d**,**e**) The side views of the goldene monolayers at the ultimate tensile strength point uniaxially loaded at 300 K along x and y directions, respectively, with color coding representing the out-of-plane displacements of atoms with respect to the center of mass.

We next explore the mechanical features of the goldene monolayer on the basis of DFT and MTP-based modeling. According to the DFT calculations, the C_11_, C_22_ and C_12_ elastic constants of the single-layer goldene are predicted to be 274, 278 and 116 GPa, respectively. Based on these values, the elastic modulus along the x and y directions is calculated to be 226 and 229 GPa, which is almost one-fourth of that for graphene. Although slightly different, these results reveal, a convincingly isotropic elasticity of the goldene nanosheet. Next, the mechanical responses were examined using uniaxial tensile loading [49,50,51,52,53,54,55,56]. In this regard, within the DFT and MTP-based methods at the ground state without the temperature effect, the conjugate gradient algorithm coupled with box relaxation in the perpendicular direction of loading was employed to ensure the accurate satisfaction of the uniaxial stress condition. In Figure 2, the uniaxial stress–strain curves of the pristine goldene monolayer uniaxially loaded along the x and y directions predicted by the DFT and the MTP-based model are compared, confirming the remarkable precision of the developed MLIP in scrutinizing the direction-dependent mechanical response of the goldene nanosheet. While the initial parts of the stress–strain curves are precisely reproduced by the validated MTP method, indicating an accurate representation of the elastic response, discrepancies with DFT results arise as the maximum tensile strength is approached. For the uniaxial loading along the x direction, with the DFT method around the maximum tensile strength point, unusual variations manifest in the stress–strain curve, inconsistent with conventional behaviors. Conversely, when loaded along the y direction, the DFT method exhibits a smooth decline in stress after reaching maximum tensile strength, whereas the MTP demonstrates a notably abrupt drop. Unconventional stress–strain curves predicted by DFT around the maximum tensile strength point can be attributed, in part, to the small unit cell and also the artifacts of DFT modeling [35]. From a modeling point of view, it is crucial that the trained model accurately reproduces the elastic response and ultimate tensile strength point, which are critical pieces of information for the design of nanodevices. The ultimate tensile strengths of the goldene monolayer along the x(y) directions are predicted to be 11.9(19.6) GPa by the DFT, which is remarkably close to the corresponding values of 11.4(19.8) GPa predicted by the MTP method. Despite some deviations in the stress–strain relations between the MTP and DFT method results, it is noteworthy that critical mechanical properties such as elastic modulus and tensile strength are accurately reproduced by the MTP method. This highlights the significance of the MTP approach in capturing key mechanical features. After ensuring the accuracy of the trained MTP, we next evaluate the mechanical properties of the goldene monolayer at 300 K by considering 1056 atoms in the simulations. In this case, quasi-static uniaxial tensile loading was employed, utilizing the Nosé–Hoover barostat and thermostat method (NPT) to satisfy the uniaxial stress conditions, as detailed in our previous studies [33,34]. The tensile strengths of the goldene monolayer at room temperature along the x(y) directions are predicted to be 9.4(12.9) GPa, which is almost an order of magnitude lower than those predicted for pristine graphene [33]. In Figure 2, we delved into the failure mechanism of the goldene nanosheet at 300 K under various loading directions. When loaded along the x-direction, it can be seen that the Au-Au bonds, which are oriented exactly along the loading direction, start to break first, leading to the formation of a series of interconnected four-membered rings, which subsequently contribute to the failure of the nanosheet. On the other hand, for uniaxial loading along the y-direction, the Au-Au bonds are originally tilted by an angle of about 30 degrees with respect to the loading direction. In this case, the breakage of the first Au-Au bonds leads not only to the formation of four-membered rings but also to the appearance of hexagonal rings prior to system failure. It is noteworthy that the formation of hexagonal rings aligns with observations from the original experimental work [26]. Based on the presented first-principles results, it is conspicuous that while the elastic response of the goldene nanosheet exhibits a convincing isotropy, the tensile strengths and failure mechanisms are clearly anisotropic, which arises due to the distinct bonding configurations prevalent under different loading directions. 

**Figure 3:**
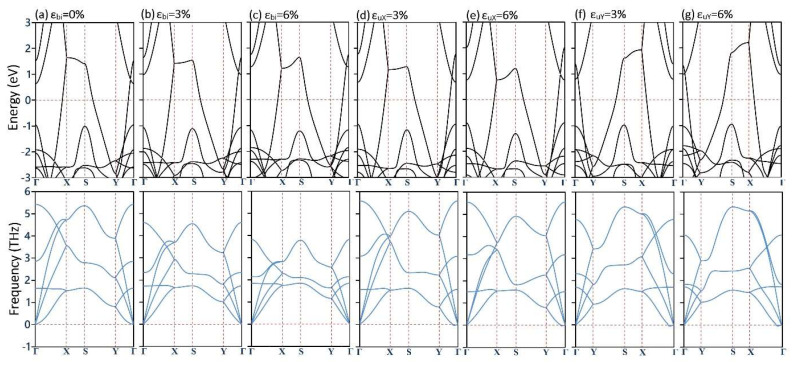
(**First row**) Electronic band structure of the goldene monolayer under different biaxial (ε_bi_) and uniaxial loading along x (ε_uX_) and y (ε_uY_) tensile strains based on the PBE functional. (**Second row**) Predicted phonon dispersions for the strained monolayers obtained by the MTP approach.

We finally examine the electronic and dynamic stability of the biaxially and uniaxially loaded goldene monolayers. In this case, we considered strain levels of 3 and 6%, which are below the failure point. The phonon dispersions are evaluated by the MTP method, which, unlike the DFPT method, demands a negligible computational cost. The results shown in Figure 3 reveal that the native metallic nature of the goldene monolayer stays completely intact under large tensile strains. It is worth mentioning that quantum confinement can affect the electronic properties of multi-layered 2D materials [10,57,58], which should also be investigated in detail for the goldene nanosheets in the upcoming studies. Moreover, the results shown in Figure 3 confirm the remarkable dynamical stability of the goldene nanosheet under various large tensile strains. As is clear, the metallic nature of the goldene nanomembranes suggests that the thermal transport in this system can be also carried by the electrons, and as such, while the lattice thermal conductivity is estimated to be low, the electronic contribution may become decent, which should be explored in the oncoming studies. 

## 4. Concluding Remarks

Inspired by the synthesis of the goldene nanosheet [26], in this communication, comprehensive first-principles calculations were carried out to explore the stability, electronic, mechanical and phononic properties of single-layer and free-standing goldene. The presented results highlight that goldene can demonstrate remarkable flexibility and dynamical stability and stay completely intact at the elevated temperature of 700 K, indicating the promising potential for practical applications. It is confirmed that the stress-free and largely strained goldene monolayer exhibits a metallic electronic nature. At room temperature, the goldene nanosheet is found to show a slightly anisotropic lattice thermal conductivity, around 10 ± 2 W/(m.K). Because of the metallic nature of goldene nanomembranes, the electronic contribution to thermal transport may become significant, resulting in a higher effective thermal conductivity. The C_11_, C_22_ and C_12_ elastic constants of the single-layer goldene are predicted to be 274, 278 and 116 GPa, respectively, confirming the decent rigidity of this system. The tensile strengths of the goldene monolayer at room temperature along the x(y) directions are predicted to be 9.4(12.9) GPa. It is shown that that while the elastic response of the goldene is convincingly isotropic, the tensile strengths and failure mechanisms are anisotropic due to different bonding along two in-plane loading directions. Presented first-principles results provide a comprehensive understanding of the stability, mechanical, thermal and electronic properties of the goldene monolayer.

## Data Availability

Please find: https://doi.org/10.17632/s3bw2vksx6.1 for the related data for this work. Additional data presented in this study are available on request from the author.
